# Heterologous Epitope-Scaffold Prime∶Boosting Immuno-Focuses B Cell Responses to the HIV-1 gp41 2F5 Neutralization Determinant

**DOI:** 10.1371/journal.pone.0016074

**Published:** 2011-01-26

**Authors:** Javier Guenaga, Pia Dosenovic, Gilad Ofek, David Baker, William R. Schief, Peter D. Kwong, Gunilla B. Karlsson Hedestam, Richard T. Wyatt

**Affiliations:** 1 Vaccine Research Center, National Institutes of Health, Bethesda, Maryland, United States of America; 2 Department of Microbiology, Tumor and Cell Biology, Karolinska Institutet, Stockholm, Sweden; 3 University of Washington, Seattle, Washington, United States of America; University of Kansas Medical Center, United States of America

## Abstract

The HIV-1 envelope glycoproteins (Env) gp120 and gp41 mediate entry and are the targets for neutralizing antibodies. Within gp41, a continuous epitope defined by the broadly neutralizing antibody 2F5, is one of the few conserved sites accessible to antibodies on the functional HIV Env spike. Recently, as an initial attempt at structure-guided design, we transplanted the 2F5 epitope onto several non-HIV acceptor scaffold proteins that we termed epitope scaffolds (ES). As immunogens, these ES proteins elicited antibodies with exquisite binding specificity matching that of the 2F5 antibody. These novel 2F5 epitope scaffolds presented us with the opportunity to test heterologous prime∶boost immunization strategies to selectively boost antibody responses against the engrafted gp41 2F5 epitope. Such strategies might be employed to target conserved but poorly immunogenic sites on the HIV-1 Env, and, more generally, other structurally defined pathogen targets. Here, we assessed ES prime∶boosting by measuring epitope specific serum antibody titers by ELISA and B cell responses by ELISpot analysis using both free 2F5 peptide and an unrelated ES protein as probes. We found that the heterologous ES prime∶boosting immunization regimen elicits cross-reactive humoral responses to the structurally constrained 2F5 epitope target, and that incorporating a promiscuous T cell helper epitope in the immunogens resulted in higher antibody titers against the 2F5 graft, but did not result in virus neutralization. Interestingly, two epitope scaffolds (ES1 and ES2), which did not elicit a detectable 2F5 epitope-specific response on their own, boosted such responses when primed with the ES5. Together, these results indicate that heterologous ES prime∶boost immunization regimens effectively focus the humoral immune response on the structurally defined and immunogen-conserved HIV-1 2F5 epitope.

## Introduction

Most effective anti-viral vaccines protect by the elicitation of neutralizing antibodies [1], [2], therefore the elicitation of broadly neutralizing antibodies to the surface-exposed HIV-1 envelope glycoprotein (Env) spike is likely a critical component for an effective HIV-1 vaccine. The trimeric spike is comprised of the highly N-glycosylated exterior Env, gp120, and the non-covalently associated transmembrane Env, gp41 and is the sole virally encoded target for neutralizing antibodies [3]. The gp120 subunit binds the host primary cellular receptor, CD4, and following receptor-induced conformational changes, the target cell co-receptor, CCR5 [4], [5], [6]. Following CCR5 engagement by gp120, gp41 mediates viral-to-target cell fusion, facilitating entry of viral genetic information into the cell and onset of retroviral infection.

During chronic HIV-1 infection, selected individuals generate broadly neutralizing antibodies to the functional Env spike [7], [8], [9], [10], [11], [12], [13], [14], [15], [16], and a subset of these responses map to conserved elements of Env [17], [18]. However, in general, the elicitation of broadly neutralizing HIV-1 antibodies following natural infection appears relatively inefficient [19], [20], [21], [22], [23]. Reflective of this inefficiency, until recently, only four broadly neutralizing antibodies isolated from HIV-1-infected individuals were described. Two of these antibodies bind to conserved epitopes in the gp120 subunit, b12 and 2G12 [24], [25]; and two bind to conserved, contiguous epitopes in the gp41 subunit, 2F5 and 4E10 [26], [27]. In the past year, several new broadly neutralizing antibodies have been described and include the trimer-preferring antibodies, PG9 and PG16, and the CD4 binding site antibodies HJ16, VRC01/2 and VRC03 [28], [29], [30].

The gp41-directed 2F5 and 4E10 antibodies target the gp41 membrane external proximal region (MPER), and are accessible at some not yet well defined juncture during viral entry, permitting MPER-directed neutralization [31], [32]. Numerous prior efforts to elicit antibodies against this gp41 region using diverse MPER-base immunogens resulted in low epitope-specific antibody titers that displayed limited, weak, or no neutralization activity [33], [34], [35], [36], [37], [38], [39], [40]. The peptide epitope conformations of the MPER-directed neutralizing antibodies are crystallographically defined in complex with the corresponding Fab fragment at the atomic level of resolution, allowing structure-guided pathways for immunogen design. A novel and recently described method for immunogen design known as “scaffolding” uses the power of computational design to engraft the 2F5 epitope in its unusual and fixed conformation onto selected unrelated, non-HIV derived protein ‘acceptor’ scaffolds [41], and similarly applied for the 4E10 epitope [42]. The 2F5 linear epitope presents a unique challenge for the scaffolding approach as it naturally tends to adopt a helical conformation as defined by NMR or by structures of the post-fusogenic conformation of gp41 [43], [44], [45], [46]. However, in complex with the 2F5 antibody, this region assumes an extended loop conformation [47], [48]. As immunogens inoculated into guinea pigs, the 2F5 ES proteins elicited antibodies with exquisite binding specificity matching that of the parental 2F5 antibody [41].

The creation of the novel 2F5 epitope-scaffold (ES) proteins suggested a strategy to focus antibody responses to the conserved 2F5 epitope by designing and inoculating in series a set of unrelated scaffolds displaying the 2F5 determinant as the only epitope in common. This approach also eliminates glycan occlusion and immunodominant gp120 variable regions, which may obscure or divert the antibody response from desired broadly neutralizing Ab determinants in gp41 or gp120. The aim of this approach is to prime B cell responses to the 2F5 epitope displayed by one of the ES proteins then to boost with unrelated ES proteins displaying the same 2F5 epitope to selectively stimulate memory B cells specific for the shared antigenic determinant.

In the current study, using selected 2F5 ES proteins, we demonstrated efficient heterologous prime∶boosting that, with each succeeding boost, increases elicitation of 2F5 epitope-specific antibodies and B cells. We observed that, in the heterologous prime∶boosting regimen, incorporation of a linked T cell helper epitope (TH) in the immunogens was advantageous to the 2F5 epitope-specific elicitation at the serum antibody binding level and at the secretory B cell level. Most importantly, we observed profound alterations in presentation of the 2F5 epitope graft to elicit B cell responses that was scaffold context-dependent. As before, we observed that only one epitope scaffold combination (ES5) efficiently elicited 2F5 epitope-specific B cells, while two others (ES1 and ES2), although they contained the 2F5 epitope and fixed conformation, poorly elicited 2F5 epitope-specific antibodies. Interestingly, following the efficient priming with ES5, the “non-epitope-presenting” ES1, ES2 proteins were then able to efficiently boost 2F5 epitope-specific B cell responses at the polyclonal and monoclonal level. However, in no case did we elicit 2F5 epitope-directed antibody responses capable of virus neutralization.

The results presented here suggest that the use of computationally designed epitope scaffolds may be useful for targeting humoral responses on structurally defined sites on viral pathogens. Specifically, the 2F5 epitope-scaffolds provide a well-defined system to evaluate and optimize immunization based upon this approach, which may be required to elicit epitope-specific responses against the well-shielded HIV-1 functional spikes.

## Materials and Methods

### Ethics Statement

Animal experiments were carried out at two separate locations, the Vaccine Research Center (VRC) at the National Institutes of Health and at the Karolinska Institutet in Sweden. All animal experiments were approved by the Institutional Animal Care and Use Committee (IACUC) at the NIH (approval number A4149-01) and by the Swedish committee Stockholm's Norra Djurförsöketiska Nämnd (approval number N475/09), and performend according to given guidelines.

### Cloning, expression and purification of 2F5 epitope scaffolds

Plasmids for expression of the 2F5 ES fusion proteins termed ES5, ES1, ES2 and ES4 were initially derived by *de novo* gene synthesis and subcloning into the mammalian expression vector, CMVR as previously described [41]. The acceptor scaffolds were based upon proteins with the Protein Data Base designation 1d3bb, 1lgya, 1ku2a and 1iwla respectively. The fusion proteins ES2 and ES4 did express in the mammalian system. However, scaffolds ES5 and ES1 did not express in the mammalian system and were subcloned into a bacterial expression vector (pET-17b, Novagen). Versions of the scaffolds ES1, ES2 and ES5 with an engineered universal heterologous T cell helper epitope (AFKVAAWTLKAAA) at the C-terminus were derived by Quickchange PCR mutagenesis (Stratagene). Mammalian expression of the proteins ES2 and ES4 was carried out in 293F cell line that has been adapted to serum-free medium (Invitrogen). In brief, the 293F cells were grown in 2L flasks to a density of 1.2×10^6^ cells per ml and transfected with 250 µgs of plasmid DNA per liter of medium using 293 Fectin transfection reagent (Invitrogen). Cell culture supernatants were collected 4 days after transfection, centrifuged at 3,500×g to remove cell debris and filtered using a 0.22 µm filter unit. Supernatants were applied to a His-Trap nickel affinity column (Amersham). The column was washed with 100 ml phosphate buffer containing 20 mM imidazole, pH 7.4 and then eluted with Phosphate buffer containing 500 mM imidazole. Eluates were concentrated and subjected to buffer exchange with phosphate buffer pH 7.4 using Amicon ultra 10,000 MWCO centrifugal filter devices (Millipore). A second round of affinity purification using a 2F5 mAb column was performed. Buffer exchanged eluate from Nickel purification was applied to the 2F5 antibody Affinity column, then washed with 100 ml of 500 mM NaCl in phosphate buffer, pH 7.4 and proteins were eluted with IgG elution buffer, pH 2.8 (Pierce). Elution buffer acidic pH was quickly neutralized with TRIS buffer, pH 8.5. Eluates were pooled, concentrated and buffered exchanged to phosphate buffer, pH 7.4.

Scaffolds in the pET-17b vector were expressed in Rosetta BL21 *E. Coli* bacteria. In brief, a 50 ml culture of transformed *E. coli* was grown overnight at 37°C. The following day a 1 L culture was grown from the overnight 50 ml culture to 0.6 OD and expression of the protein was induced by using IPTG at a final concentration of 1 mM, then the culture was grown for 6 more hours. Bacteria were pelleted by centrifugation at 5,000×g and protein was extracted from inclusion bodies. First, bacterial cell pellets were lysed using Novagen Bugbuster reagent containing lysozyme. Lysate was subjected to centrifugation at 10,000×g for pelleting inclusion bodies. Isolation of the proteins from inclusion bodies was carried out using denaturing conditions of 8 M urea and 1 mM ß-mercaptoethanol. After filtering insoluble debris, soluble denatured protein was purified by Nickel column in denaturing conditions (8 M urea, 10 mM imidazole) and concentrated to a volume of 1 ml using the Amicon Ultra (Millipore). Protein refolding was done at 4°C by quick dilution (1/100) into an appropriate refolding buffer and incubating at 4°C for 16 hrs. Refolding buffer contained 50 mM Tris pH 8.0, 250 mM NaCl, 500 mM L-Arginine, 0.1 mM glutathione reduced, 0.01 mM glutathione oxidized, 0.03% N-lauryl-sarcosine and 0.1 mM zinc chloride. The refolded proteins were then concentrated to approximately a volume of 5–7 mL using centricon plus-80 (Millipore) and dialyzed to PBS 125 mM NaCl using a dialysis cassette (Pierce). A second round of purification using a 2F5 antibody affinity column was carried out for ES5. However, ES1 was only Nickel purified due to insolubility at acidic elution conditions.

### SPR kinetic binding analysis

All kinetic reactions were performed at RT on a Biacore 3000 surface plasmon resonance spectrometer. To prepare binding surfaces with approximately 500 RU per cell, 10 µg/ml of ligand 2F5 antibody in 10 mM NaOAc, pH 5.5 buffer were immobilized on a Biacore CM5 chip by the amine coupling method following manufacturer's protocol. The reference cell received only NaOAc buffer. Analytes were serially diluted in the HEPES-EP reaction buffer at concentrations ranging from 3.9 nM to 500 nM for ES5, ES2 and ES4, and from 62.5 nM to 2000 nM for ES1. Association was allowed for 3 min at 30 µL/min. Dissociation was determined by washing off bound analyte over the next 3 minutes. The chip surface was regenerated with two injections of 10 mM glycine, pH 3.0 for 30 seconds. The kinetic rate constants were obtained by fitting the curves to 1∶1 Langmuir binding model using BIAevaluation software. SPR kinetic analysis of the murine monoclonals was done following the same format, immobilizing the IgG on a CM5 chip. The analytes ES2 and ES4 were run in the same concentrations as used for the 2F5 antibody analysis. For this study, we used a modified version of the gp41 MPER peptide to enhance solubitiy and facilitate binding detection (EQELLELDKWASLGGGGSGGWNWFDITKWLWYIKKKKGSKKK). This peptide was used as an analyte in a concentration series ranging from 3.9 nM to 250 nM for both the 2F5 and the murine monoclonal antibody SPR kinetic analysis.

### ELISA

200 ng/well of antigen was incubated overnight at 4°C in wells of a Maxisorp high binding plate (Nunc) in PBS, pH 7.4. The next day plates were washed five times with PBS, pH 7.4 containing 0.2% Tween 20 and blocked with 300 µL per well of PBS, pH 7.4, supplemented with 2% dry-milk powder (Difco) and 5% heat-inactivated Fetal Bovine Serum (Sigma or Gibco) for 2 hrs at RT. Plates were washed five times with PBS, pH 7.4, 0.2% Tween 20. Serum was serially diluted fivefold (1∶50 to 1∶781,250) in PBS pH 7.4, 0.2% Tween 20 and incubated for 1 hr at RT. Plates were washed five times with PBS pH 7.4, 0.2% Tween 20 and a goat secondary anti-mouse immunoglobulin G (H+L) (Jackson Labs) was incubated at a 1∶10,000 dilution in PBS pH 7.4, 0.2% Tween 20 for 1 hr at RT. Plates were washed five times with PBS pH 7.4, 0.2% Tween 20 and 100 µl of colorometric TMB (3,3′, 5, 5′-tetramethylbenzidine) peroxidase enzyme immunoassay substrate (Bio-Rad) was added to each well, and the reaction was stopped by adding 100 µl of 0.1 N H_2_SO_4_ to each well. The optical density was read on a microplate reader (Molecular Devices) at 450 nm using Softmax software.

### Animal immunizations and cell preparation

Adult female C57BL/6 mice (The Jackson Laboratory) received three inoculations subcutaneously with 20 µg of protein formulated in 10 µg AbISCO®-100 adjuvant (Isconova AB) at two weeks intervals. Pre-bleeds prior to first inoculation as well as bleeds were collected 7–10 days after each inoculation. Serum was incubated at 55°C for 1 hr to heat-inactivate complement and stored at −80°C until subjected to analysis. Three or four days after inoculation, single cell suspensions were prepared from spleen and lymph node (LN) by passing the tissue through a nylon mesh. Red blood cells were lysed with hypotonic ammonium chloride solution. After washing, the cells were resuspended in complete RPMI medium containing 5% FCS, 50 µM β-mercaptoethanol, 2 mM L-glutamine, 100 U/ml penicillin and 100 µM streptomycin (all from Sigma). Cells were then added to ELISpot plates immediately or stimulated *in vitro* at a concentration of 1×10^6^ cells/ml in complete RPMI medium containing 2 µg/ml LPS (Sigma) and 0.5 µg/ml CpG ODN 1826 (TriLink BioTechnologies) for 6 days. The *in vitro* stimulation allows for memory B cell expansion and differentiation into antibody-secreting plasma cells that can then be detected in the ELISpot assay.

### B cell ELISpot assays

The procedure and optimization of the B cell ELISpot assay was described in detail elsewhere [49]. Briefly, 96-well MultiScreen-IP filter plates (Millipore) were pre-treated with 70% ethanol and washed 3 times in sterile PBS, before coated with 1 µg/well of a polyclonal goat anti-mouse IgG antibody (Mabtech AB). The plates were incubated overnight at 4°C. Before addition of the cells, plates were washed 5 times in sterile PBS and then blocked with complete RPMI medium at 37°C for 2 hrs. Cells were added in duplicates to the wells in 3-fold serial dilutions, starting at 2,000 cells/well for hybridoma cells, 1×10^6^ cells/well for splenocytes and 1.3×10^6^ originally cultured cells/well for the *in vitro* stimulated cells. Plates were wrapped in plastic and incubated for 12 hrs at 37°C. For detection of spots, the cells were removed by washing the plates 6 times in PBS containing 0.05% Tween 20. For detection of total IgG secreting cells, 0.1 µg/well of a biotinylated polyclonal goat anti-mouse IgG (Mabtech AB) was added in blocking buffer (PBS containing 1% FCS and 0.05% Tween 20). For detection of graft-specific B cells, biotinylated 2F5 peptide (EQELLELDKWASLW) (0.1 µg/well) or control protein β-galactosidase (0.2 µg/well) were added as probes diluted in blocking buffer. Biotinylated probes were incubated in the plates for 2 hrs at room temperature (RT). Responses to protein epitope scaffold used as immunogens were measured with unbiotinylated probes. In this case, an additional incubation step with a rabbit anti-his tag antibody (0.2 µg/well) (Immunology Consultants Laboratory, Newberg OR) was performed. Plates were then subjected to 6 washes of PBS containing 0.05% Tween 20 before addition of 100 µl of alkaline phosphatase (ALP)-conjugated streptavidin (Mabtech AB) diluted 1∶1000 in PBS. Plates were incubated for 45 min at RT and then washed 6 times in water. 100 µl of BCIP/NBT-plus substrate (Mabtech AB) was added and incubated for 10 min at RT. Plates were then washed extensively with water and air-dried. Spots were counted in an ImmunoSpot^R^ analyzer (Cellular Technology Ltd.).

### Epitope-scaffold elicited mouse monoclonal antibodies

As previously described [41], Balb/c mice were inoculated subcutaneously with 20 µg of protein in Alum and CpG combination adjuvant following either a homologous regimen of 5 inoculations of ES5 or a heterologous prime∶boost regimen of two inoculations of ES5 followed by 3 inoculations of ES1. For this study, we included analysis of monoclonals derived in parallel from mice inoculated 5 times with the ES1 immunogen and isolated and characterized as previously described [41]. In brief, ELISA IgG titers measured using heterologous ES2 protein or (EQELLELDKWASLWNWFDITKWLWYIKKKKGSKKK) gp41 MPER peptide were used to determine mice that were to be sacrificed to proceed with fusion of splenocytes to generate hybridoma cells. 2F5 epitope-specific clones were selected on ES2, ES3 and ES4 epitope-scaffolds. Here we report a comparative binding analysis of the new ES1-derived monoclonals (14B, 14E and 5C1) and the previously described monoclonals (1D9, 1C1, 9F8 and 11F, 6A7, 6F4).

## Results

### Biophysical characterization of the 2F5 epitope-scaffolds (ES)

**Figure 1:**
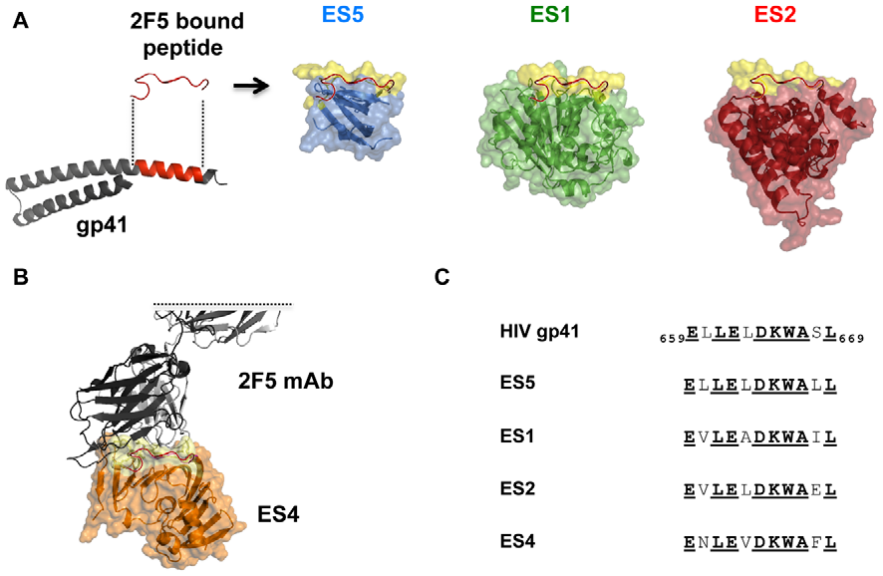
The 2F5 epitope scaffold (ES) fusion proteins. (A) Left, in red the 2F5 gp41 epitope region is shown both in the post-fusogenic helical form (PDB 3K9A) and in the 2F5-bound conformation (PDB 1TJI). The gp41 membrane proximal external region (MPER) including the 2F5 epitope adopts most frequently an alpha-helical conformation, however, it forms an extended ß-turn loop conformation when bound to the 2F5 antibody, as described in [48]. Structural models (pymol) of the ES proteins used as immunogens ES5 (blue), ES1 (green) and ES2 (red), respectively. Their molecular surfaces are rendered translucent to display the underlying secondary structure. Superimposed (in red) is the 2F5 antibody-bound peptide conformation. The conserved 2F5 epitope graft molecular surface is shown in yellow. (B) Partial structure of the 2F5 antibody Fab (gray) docked to the model of ES4 (orange). ES4 was used as an antigenic probe to measure epitope-specific responses to the conformationally constrained 2F5 epitope and was not used as an immunogen. (C) Alignment of the gp41 2F5 epitope and the ES graft sequences; the 2F5 antibody contact residues defined in the 2F5 antibody-peptide structure are emboldened and underlined.

For the serial prime∶boost immunogenicity analysis that was the focus of this study, we characterized a subset of the previously described epitope-scaffolds, namely ES1, ES2, ES4 and ES5 (see Fig 1 for schematic fusion protein models and graft sequences). Because several of the ES are relatively short in linear sequence, and might be deficient in T cell helper epitopes, all of the immunogen proteins were designed either lacking or possessing a promiscuous, heterologous T cell helper epitope at the C-terminus (previously called TH, similar to PADRE, [50]).

**Figure 2:**
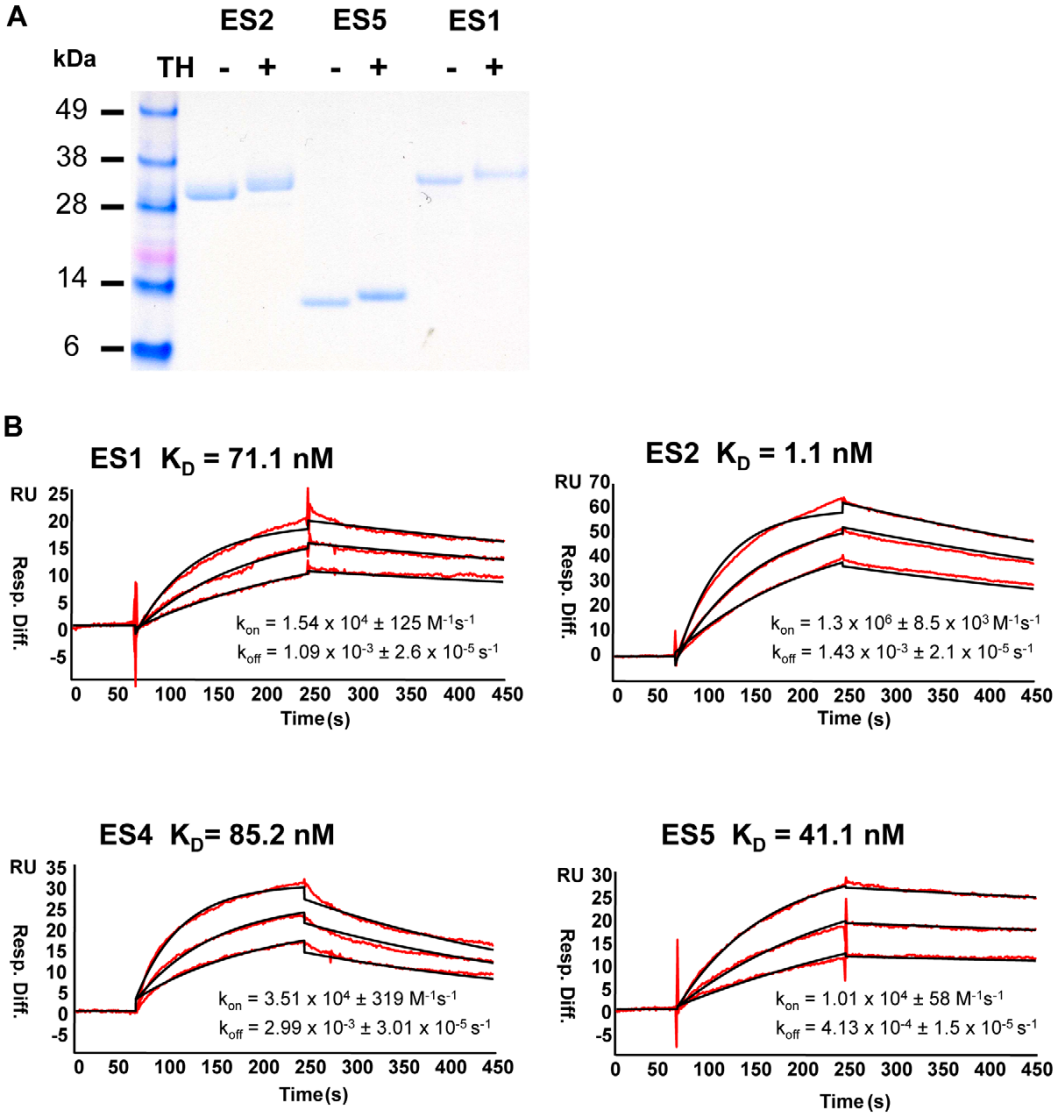
Biophysical characterization of the ES proteins. (A) SDS-PAGE gel of ES proteins used as immunogens after affinity purification. The ES proteins possessing the C-terminal heterologous T cell helper residues are denoted “+TH”. (B) The recognition of the ES proteins by the 2F5 monoclonal antibody was assessed by surface plasmon resonance (SPR) in a Biacore 3000 instrument. In red, the observed data obtained by flowing the ES proteins as analytes over a CM5 chip to which the 2F5 IgG antibody was immobilized. In black, fit curves when a 1∶1 Langmuir model is applied to the observed data. Affinity constant values are indicated above the curves and the rate constants are denoted below the curves.

Following expression and purification, the ES1, ES2 and ES5 immunogens, either lacking or possessing the promiscuous TH, were determined to be relatively homogenous by SDS PAGE (Fig 2A). In each case, the TH-containing epitope scaffolds migrated more slowly in the gel, consistent with the presence of the 13 residue, TH, C-terminal adduct. Binding recognition of the ES proteins by the 2F5 monoclonal antibody was determined both by Surface Plasmon Resonance (SPR) (Fig 2B) and by ELISA (data not shown). The affinity of 2F5 for each of the ES proteins was determined by immobilizing the 2F5 antibody to the surface of the SPR chip and flowing the ES proteins as solution analytes over the surface. In this configuration, the affinity of 2F5 for the ES2, ES5, ES1 and ES4 analytes was determined to be 1.1, 41.1, 71.1 nM and 85.2 nM, respectively. Some of these values differ slightly from our previous report, in which case the ES proteins were attached directly to the solid phase SPR chip, and the 2F5 antibody Fab was configured as the analyte [41]. These are relevant affinity differences to report as they might be relevant to the differential presentation of the 2F5 epitope to the immune system in selected scaffold contexts. It is likely that the observed “apparent affinity differences” between the two configurations may be indicative of some oligomers of the ES proteins in solution, resulting in a partial occlusion of the 2F5 graft as well as avidity gain that could influence the observed apparent affinity. In any case, this alternative and new SPR analysis revealed that the ES5 protein, showed considerably slower dissociation rates (4.13×10^−4^ s^−1^) as compared to the rates of other ES proteins, which were 5 to 10 times faster in their off-rates (Fig 2B). The slower observed dissociation rate may be a result of oligomerization of the ES5 protein in solution and may contribute to its ability to enhance anti-2F5 epitope responses in our present (and past) immunogenicity study. By this analysis, the 100-fold more rapid on-rate of 2F5 to the ES2 protein was consistent with our previous observation that the 2F5 epitope may be more tightly locked into the extended loop conformation [41]. Presumably, this conformational constraint was accomplished by underlying protein-protein interactions, as modeled during the design of this epitope-scaffold combination.

### Immunogenicity of ES in a homologous regimen

**Figure 3:**
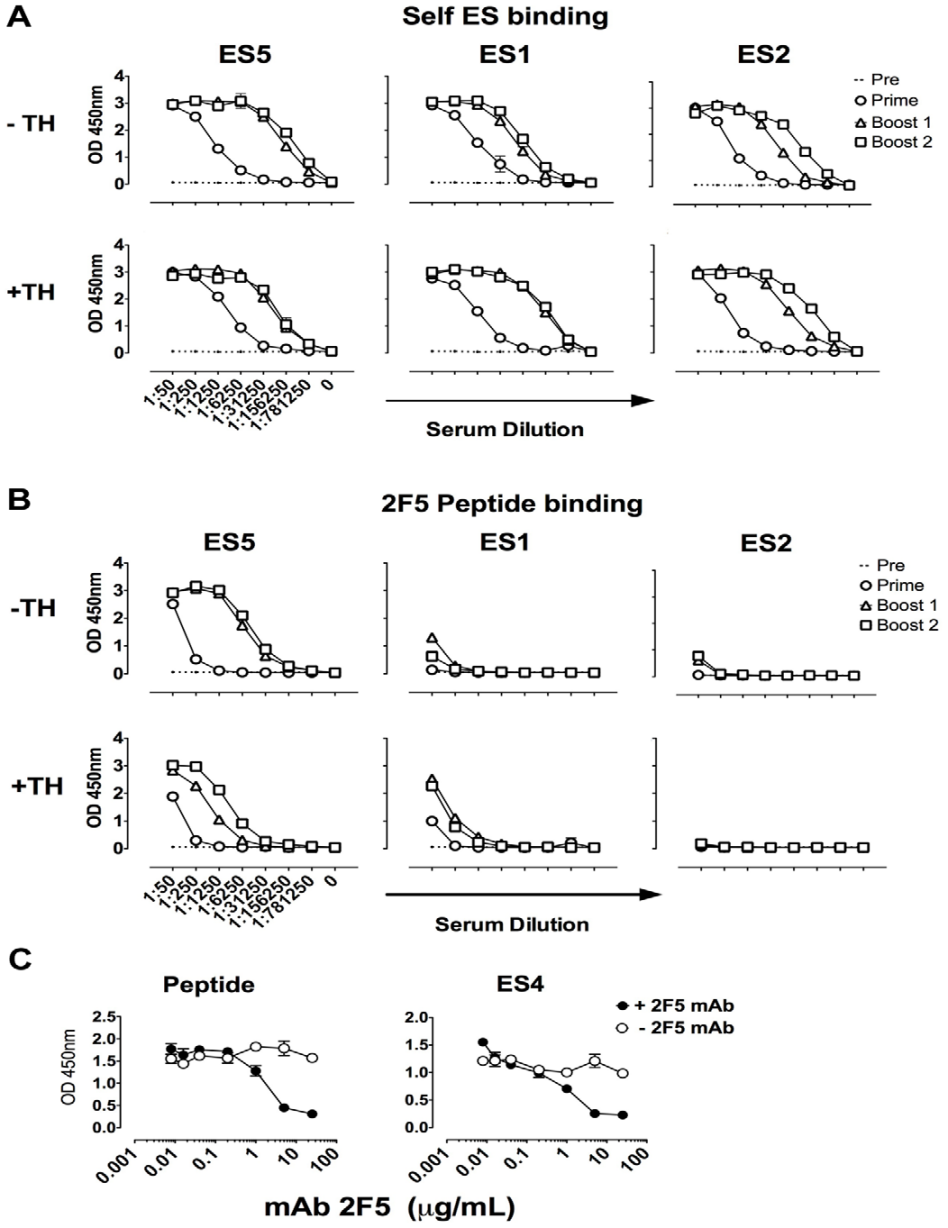
ELISA binding results of ES-elicited sera following a homologous inoculation regimen. (A) Anti-ES titers in serum of inoculated mice (5 mice per group); upper panels depict titers obtained after immunizations with constructs not possessing the T cell helper epitope TH, and are denoted as “−TH”; bottom panels corresponds to immunizations of constructs possessing the T cell helper epitope and are denoted as “+TH”. (B) ES-elicited serum binding titers measured against the 2F5 peptide adsorbed to the ELISA plate. (C) Competition between ES5-elicited serum (at a 1∶2000 dilution) and the 2F5 mAb (serial concentrations) for binding to peptide (left) and ES4 (right) absorbed to the ELISA plate. Open circles represent binding of ES5 sera in the absence of the 2F5 mAb competitor and closed circles represent binding of the ES5 sera in the presence of increasing amounts of the competitor 2F5 mAb.

We first analyzed the responses in animals inoculated subcutaneously with 20 µg of the proteins ES1, ES2 and ES5 in Abisco-100 adjuvant using a homologous immunization regimen of three immunizations, two weeks apart. We determined that in the three homologous regimens performed in parallel, all three ES proteins were relatively immunogenic in the C57BL/6 mice, consistent with our recent experiments performed in outbred guinea pigs [41].The elicited antibody binding titers to each ES protein saturated after three inoculations, with relatively high endpoint titers observed (i.e., 1∶781,250 endpoint titers for ES5, ES1 and ES2) (Fig 3A). These ES-recognizing serum antibody responses are directed to the entire surface of each respective inoculated ES (blue, red and green surfaces for each ES respectively, along with the yellow 2F5 epitope graft; see Fig 1A). As seen in Fig 3A, the presence of the heterologous TH was not required for any of the ES proteins to be immunogenic when inoculated in a homologous regimen.

Next we sought to assess the capacity of the ES immunogens to elicit antibody responses specific for the 2F5 epitope using two different 2F5 epitope targets. Note that, here, we are measuring only the serum antibody response directed exclusively to the 2F5 epitope graft (yellow surface on the pymol models in Fig 1A). First, we measured binding to the free 2F5 peptide captured on the ELISA plate (Fig 3B). Second, and in parallel we measured binding to the ES4 protein target captured on the plate (Fig S1). Recall that ES4 was not inoculated into the mice and displays the bound conformation of the 2F5 epitope, and has no sero-cross reactivity with any of the other acceptor scaffolds. Therefore, the ES4 target is recognized only by graft-specific antibodies specific for the 2F5-bound conformation of the epitope. Using these two probes, we observed that, after three inoculations, ES5 elicited near saturating levels of 2F5 epitope-specific responses with endpoint titers of 1∶156,250 to the 2F5 epitope peptide and ES4 (Fig 3B and Fig S1). Furthermore, the binding of the ES5-elicited serum antibodies to both peptide and ES4 was confirmed to be specific using a 2F5 antibody cross-competition assay, as shown in Fig 3C. In contrast to ES5, the ES1 immunogen required three inoculations to elicit very low, but detectable responses directed toward the 2F5 epitope and displayed low endpoint titers of 1,250. A third pattern of responses was observed in the ES2 context, as the 2F5 epitope graft was virtually non-immunogenic in this context (Fig 3B). This extremely inefficient elicitation of B cell responses to the ES2-presented 2F5 epitope may be related to its apparent greater degree of conformational fixation as deduced from isothermal titration calorimetric analysis of the interaction between ES2 and the 2F5 antibody [41].

The presence of the heterologous TH did not greatly enhance the ability of the ES immunogens to elicit 2F5 epitope-specific responses in a homologous regimen. In fact, TH in the context of homologous ES5 prime-boosting had a slight inhibitory effect on antibodies elicited to the 2F5 epitope, perhaps by competing for class II presentation in some not yet defined manner.

### Heterologous prime∶boosting at the serum and monoclonal antibody level

We sought to determine if heterologous prime∶boosting could focus the B cell response toward the commonly shared 2F5 epitope, especially on scaffolds that do not efficiently present the 2F5 epitope to the humoral immune system on their own. This could be advantageous for scaffolding or other structure-guided immunogen approaches where, perhaps, a less fixed conformation could first prime a B cell response, and then, a more conformationally fixed, and superior structural mimetic, might drive the desired subset of memory B cells to a desired epitope. As in the classic hapten-carrier immunogenicity experiments [51], [52], the goal was to induce anti-hapten antibody titers with disregard to the protein carrier, here we aimed at eliciting anti-gp41 2F5 epitope responses, disregarding the responses to the protein scaffold that carries the epitope-graft on its surface. In principle, this might better elicit antibodies against a more highly constrained, but less immunogenic, neutralizing determinant.

**Figure 4:**
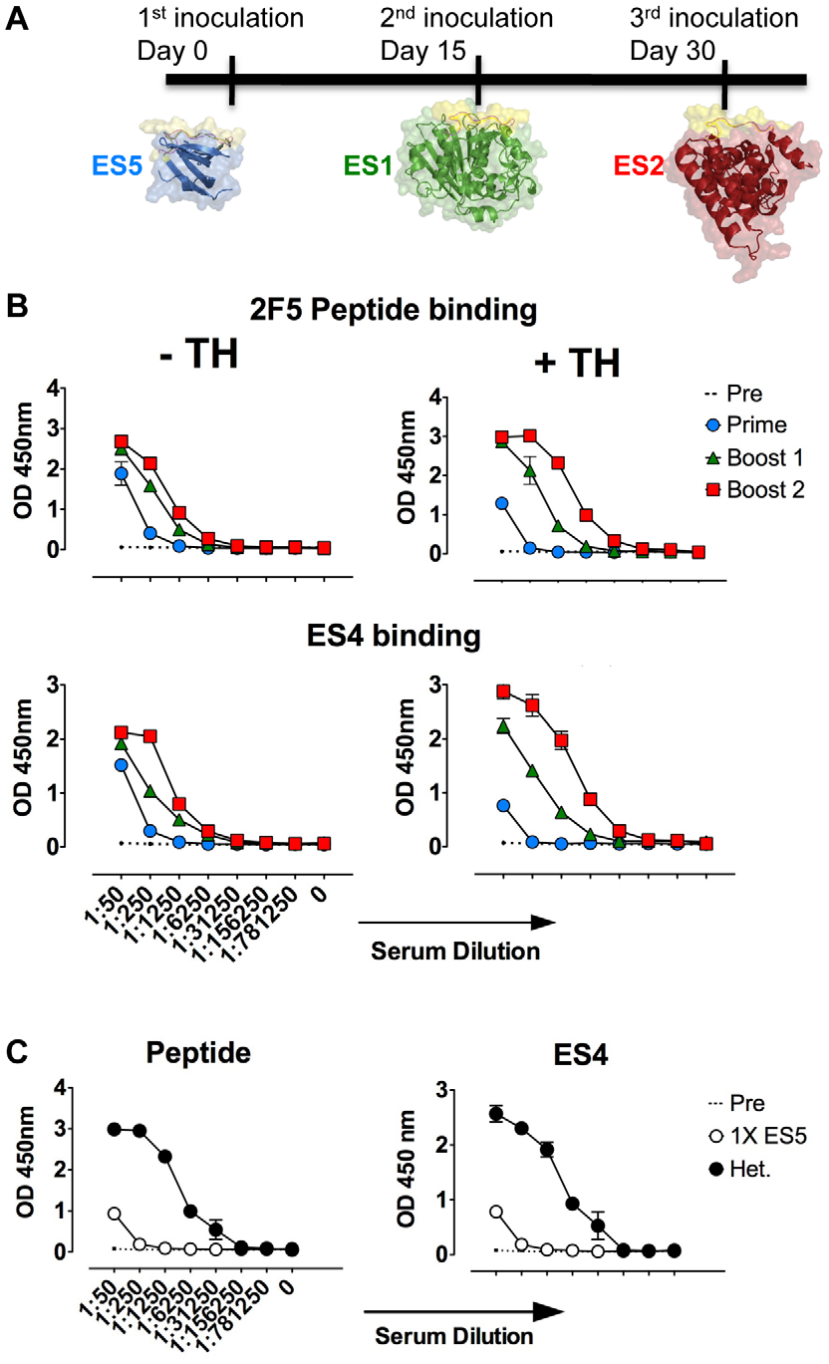
Heterologous prime∶boost regimen ELISA titers. (A) Schematic representation of the heterologous prime∶boosting regimen. (B) 2F5 peptide (top) and not inoculated ES4 (bottom) binding serum titers of pooled sera from 5 mice following a heterologous prime∶boost immunization regimen (ES5-ES1-ES2) with or without T cell helper epitope (+/−TH). (C) To confirm that the epitope specific responses obtained in the heterologous regimen were not a result of the first (priming) inoculation with the immunogenic ES5 protein, which then increase over the time of the experiment, we inoculated 5 mice once with ES5 and measured sera binding titers after the same time interval of the complete heterologous prime-boosting regimen (34 days).

We selected the order of ES5 to ES1 to ES2 based upon the relative immunogenicity of the graft as observed in both guinea pigs and mice and evaluated ES both lacking and containing linked heterologous T cell help. In this regimen (Fig 4A), each ES was inoculated once to easily determine the origin of the antibody response. The results in Fig 4B demonstrated that heterologous prime∶boosting amplified the response to the 2F5 epitope graft, as shown using both the 2F5 epitope peptide and the non-inoculated ES4 protein as coated target antigens in the ELISA. Quite interestingly, the ES2 epitope-scaffold, which elicited virtually undetectable antibodies to the 2F5 epitope as either a prime or a boost in the homologous setting, efficiently boosted responses specific for the 2F5 epitope once primed by the highly immunogenic ES5 protein (Fig 4B). ES5 also primed for a substantial increase in 2F5 epitope antibodies when boosted by ES1. The presence of the TH sequence enhanced the graft-specific responses in the heterologous prime∶boost setting of ES5 prime, followed by ES1 boost 1, followed by ES2 boost 2. In this instance, the 2F5 epitope-specific endpoint titers reached 1∶31,250 similar to the levels achieved by homologous ES5+TH inoculation, but lower than the levels elicited by ES5 lacking heterologous help (compare Fig 4B right panel to Fig 3B lower panel to Fig 3B upper panel). In contrast, less efficient heterologous prime∶boosting of responses using the isogenic constructs lacking linked T cell help was observed, with 2F5 epitope-specific endpoint titers decreased to 1∶6,250 (Fig 4B).

These serological responses described above indicated that the 2F5 epitope-specific antibody responses were elicited from established 2F5 epitope-specific memory B cells in the heterologous ES regimen, as titers to the 2F5 epitope were increased after the ES1 and ES2 boosts. Furthermore, at the conclusion of the regimen, total 2F5 epitope-specific titers were substantially higher in the heterologous regimen than those elicited by a single ES5 inoculation examined over the same time interval, confirming that the 2F5 epitope-specific titers were not simply a result of priming with the immunogenic ES5 epitope-scaffold, followed by time-dependent increases in titer (Fig 4C). Taken together, these data suggest that boosting of 2F5 epitope-specific memory B cells occurred in the heterologous prime∶boost regimen. Despite the efficient boosting of 2F5 epitope-specific responses, neither the homologous nor the heterologous regimens resulted in the elicitation of neutralizing antibodies that could be confirmed to be MPER-specific by the previously described 2F5 epitope-peptide inhibition of neutralization [53].

To assess if an advantage of heterologous prime∶boosting could be seen by an alternative means of analysis, monoclonal antibodies (Mabs) were generated from mice immunized by either a homologous regimen, comprised of 5 inoculations of ES5 or ES1, or a heterologous regimen consisting of 2 inoculations of ES5, followed by 3 inoculations of ES1. Recall that because of its poor presentation of the 2F5 epitope, 5 inoculations of ES1 were required to elicit an anti-2F5 epitope response of enough magnitude to proceed with isolation of ES1-elicited Mabs. As previously reported, the Mabs displayed similar binding specificity as the parental, human 2F5 antibody. Structural studies of two of the Mabs (11F and 6A7) showed that they induced the same ß-turn extended loop conformation when bound to the 2F5 peptide as the 2F5 antibody itself [41]. In the heterologous regimen, consisting of ES5 followed by ES1 inoculation, three booster inoculations of ES1 generated a 2F5 epitope-specific response that allowed the isolation of Mabs. As determined by SPR analysis (Table 1 and [41]), the Mabs derived from the heterologous regimen (ES5 primed, ES1 boosted) mice demonstrated higher affinity to the conformational probes (ES4 and ES2) than the Mabs generated from the homologous regimens of ES5 alone or ES1 alone. The affinity of ES1-elicited Mabs values was closer to the MAbs elicited by heterologous ES5+ES1 immunization, which is consistent with the generation of an ES1-biased memory response when the ES5-primed memory response to the 2F5 graft is driven by ES1 boosting.

**Table 1:** Mab binding kinetic constants determined by surface plasmon resonance.

		Inmunogen:		ES5			ES1			ES5 prime;ES1 boost		
		Ligand Mabs:	2F5	1D9	9F8	1C1	14B	14E	5C1	6A7	11F	6F4
**Analyte**	**Peptide** *	**K_D_ (nM)**	**3.6**	**35.8**	**80.5**	**241**	**49.3**	**43**	**44.9**	**29**	**28**	**29**
		K_on_ (1/Ms) 10^4^	71.1	120	87.9	13.6	95.2	114	96.3	197	129	117
		K_off_ (1/s) 10^−3^	2.5	42.8	70.8	32.9	47	48.9	43.2	57	37	34
	**ES2**	**K_D_ (nM)**	**2.6**	**563**	**2070**	**2020**	**94.5**	**74.9**	**82.5**	**87**	**71**	**71**
		K_on_ (1/Ms) 10^4^	95	9	1.9	0.4	105	103	87.4	134	102	99
		K_off_ (1/s) 10^−3^	2.5	51	39.3	8.6	99.7	77.3	72.1	117	73	71
	**ES4**	**K_D_ (nM)**	**85.2**	**2460**	**2380**	-	**362**	**360**	**341**	**1040**	**215**	**213**
		K_on_ (1/Ms) 10^4^	3.51	8.7	8.9	-	3.9	3.4	4	4.28	7.58	7.53
		K_off_ (1/s) 10^−3^	2.99	216	212	-	14.2	12.3	13.7	44	16	16

*peptide sequence EQELLELDKWASLGGGGSGGWNWFDITKWLWYIKKKKGSKKK.

-indicates no detectable binding.

### Analysis of responses to the 2F5 epitope graft at the B cell level

**Figure 5:**
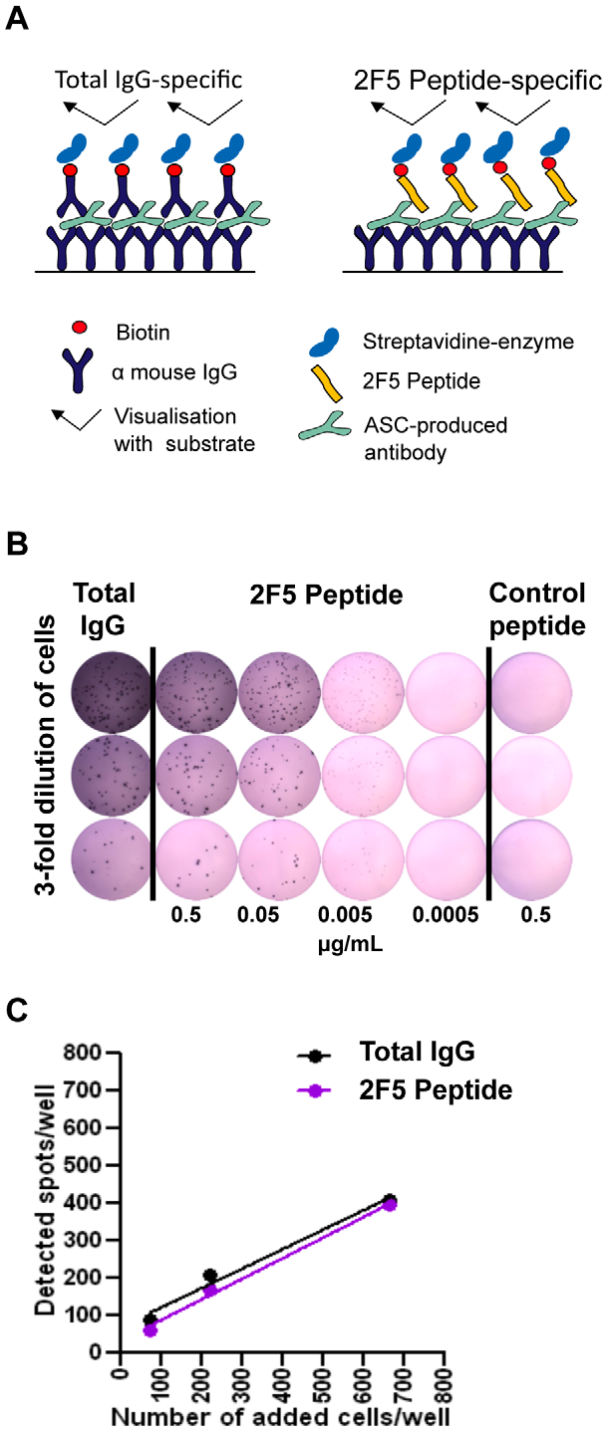
Validation of the B ELISpot using the biotinylated 2F5 peptide as a probe to measure epitope specific antigen secreting cells (ASC). (A) Schematic depiction of the modified B cell ELISpot assay [49] where all secreted antibodies are captured by rabbit anti-mouse IgG, then a biotinylated anti-mouse IgG probe is used to determine total IgG responses or a biotinylated 2F5 peptide probe is used to determine the 2F5 peptide-specific ASC. (B) ELISpot plate showing immuno spots generated by the hybridoma cells expressing the murine monoclonal antibody 1D9 [41] which binds the 2F5 epitope. (C) As shown by the 1∶1 correspondence, the 2F5 peptide probe binds nearly 100% of the hybridoma secreted antibodies.

To determine the frequency of 2F5 epitope-specific B cells stimulated by the homologous and heterologous prime∶boosting regimens, we established a 2F5 epitope-specific B cell ELISpot assay. By capturing all IgG-secreting cells and detecting antigen-specific spots with biotinylated probes an increase in resolution and less non-specific background was observed [49]. To first confirm the specificity of the 2F5 epitope-directed B cell ELISpot assay, we utilized a 2F5-like murine monoclonal antibody cell line 1D9. This hybridoma was generated by standard hybridoma fusion from mice inoculated with a regimen using the ES5 protein in adjuvant [41]. The hybridoma was selected by screening their secreted antibodies by binding to heterologous ES proteins (i.e., ES2, ES4), thus only selecting cells with 2F5 epitope-graft specificity. In brief, hybridoma cells were plated at selected densities to ELISpot plates coated with an anti-mouse-IgG polyclonal rabbit IgG and specific antibody was detected using biotinylated 2F5 epitope peptide followed by strep-avidin-HRP (Fig 5A). Hybridoma cells were plated at three different concentrations and a titration of the biotinylated 2F5 peptide was carried out to optimize the signal. An irrelevant peptide of similar length was used at highest concentration (0.5 ug/mL) as a negative control (Fig 5B). Recognition was achieved by the biotinylated 2F5 epitope peptide probe of secreted IgG “spots” from the 1D9 2F5-epitope-specific hybridoma cells for essentially all antibody secreting cells, validating probe specificity (Fig 5C).

**Figure 6:**
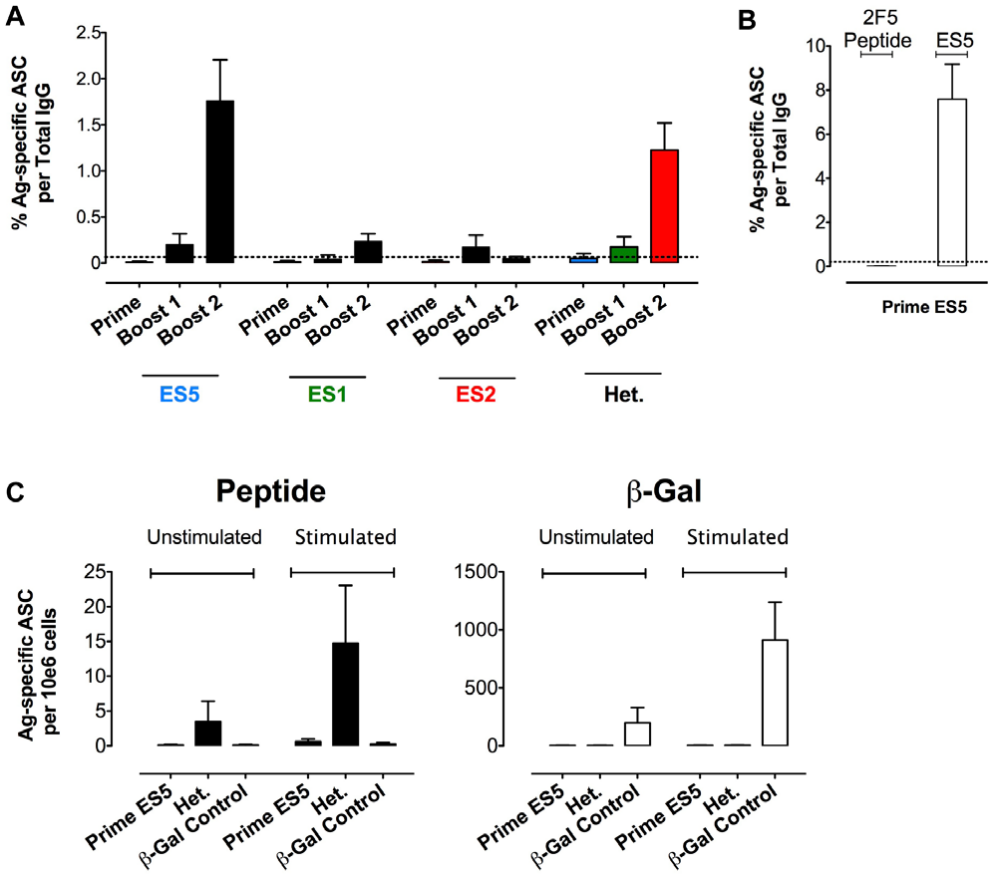
B cell ELISpots measuring epitope-specific or antigen-specific cells. (A) 2F5 peptide-specific B cell responses induced by a homologous or a heterologous regimen after each immunization. Bars represent the mean and SEM values corresponding to measurements of five mice. Colored bars represent B cell responses from the heterologous prime∶boost immunization regimen and are color-coded to indicate the immunogen inoculated prior to collection of the B cells. As a negative control we measured anti-β-Gal protein responses, which are depicted in this graph as an average of responses of all animals participating in this analysis after 3 inoculations and is plotted as the horizontal dotted line across the bars. (B) B cell ELISpot measuring peptide, anti-ES5 responses in the one-time inoculated ES5 control mice after 34 days, the length of the entire regimen. (C) 2F5 peptide-specific (left) and protein control β-Gal (right) memory B cell responses after 6 days in vitro culture in the presence (stimulated) or absence (unstimulated) of LPS stimulation. Similarly, we compare the heterologous regimen (ES5-ES1-ES2) to the one inoculation ES5 control to show that the 2F5 peptide specific memory B cells in the LPS stimulated experiment were generated via an effective cross-priming heterologous ES boost.

Having confirmed detection of 2F5 epitope-specific responses by the B cell ELISpot assay, we inoculated 15 mice per group (sacrificing 5 mice at each time point to collect B cells) with the epitope-scaffolds in adjuvant to assess B cell responses elicited by regimens analogous to those analyzed at the level of circulating antibodies. Epitope-scaffolds were inoculated either in a homologous manner for each fusion protein (i.e., ES5 prime, followed by two ES5 boosts) or in a heterologous sequential manner (ES5, followed by ES1 and ES2 sequential boosts). To ensure that immunogen-linked T cell help was functional, we inoculated ES containing the C-terminal heterologous TH sequences into C57BL/6 mice containing the I-A^b^ class II molecules, which the TH epitope binds with high affinity. 2F5 epitope-specific B cell responses, detected using the biotinylated 2F5 peptide, were detected after 3 inoculations in the ES5 homologous and the heterologous prime∶boosting groups with mean values of 1.75% and 1.22% antibody-secreting cells (ASC) of total IgG secreting cells, respectively (Fig 6A). These values were significantly higher than negative control responses to control protein ß-gal and significantly higher than those obtained after a single ES5 inoculation control animals (Fig 6B). The percentages of 2F5 epitope-specific ASC stimulated by ES5 homologous compared to the heterologous prime∶boost are of the same magnitude (not statistically significant) suggesting that the magnitude of the 2F5 epitope specific ASC response in the heterologous regimen is originating from effective ES1 and ES2 boosts that followed the ES5 prime inoculation. Notably, neither ES1 nor ES2 elicited 2F5 epitope-specific B cell responses in the homologous format after 1, 2 or 3 inoculations (Fig 6A).

To assess the presence of 2F5 epitope-specific memory B cells in the spleen, we performed the B cell ELISpot assay on splenocytes collected 3 days after the third immunization and cultured *in vitro* in the presence or absence of LPS (i.e., stimulated or unstimulated, Fig 6C). Following 6 days *in vitro* incubation with LPS to allow for proliferation and differentiation of antigen-experienced memory B cells into plasma cells, we observed a greater number of 2F5 epitope-specific spots, indicating that epitope-specific memory B cells were generated. The heterologous prime∶boost group showed a greater expansion than a single ES5 inoculation, suggesting that the recall response in the heterologous regimen is the result of an amplification of the 2F5 epitope-specific response obtained with an effective prime∶boost and not just the result of the ES5 initial priming inoculation. For the heterologous group, in cells not stimulated with LPS, less than 5 2F5 epitope-specific B cells per million cells plated were detected whereas, with LPS stimulation, approximately 15 cells per million cells plated were detected (Fig 6C, left panel). In ß-gal inoculated control mice, the antigen-specific B cell levels increased from approximately 198 in the absence of LPS to 911 in the presence of LPS (Fig 6C, right panel). The greater frequency of memory B cells specific for ß-gal likely reflects that this is a large protein, not just a single epitope as in the case of the engrafted 2F5 epitope. Consistent with this interpretation, we observed considerably more spots when we quantified B cells directed against the complete ES proteins compared to against the 2F5 epitope alone (Fig 6B and not shown).

## Discussion

In this study we investigated the potential advantage of using heterologous prime∶boosting to focus the B cell response on a structurally defined, conformational, continuous neutralizing HIV-1 Env determinant known as the 2F5 epitope. We demonstrate that, consistent with our earlier studies on this subset of scaffolds, only the ES5 protein efficiently presents the 2F5 epitope “graft” in an immunogenic manner at both the serum antibody and B cell level. In contrast, although the ES1 and ES2 constructs themselves are immunogenic overall, they do not efficiently present the 2F5 epitope graft to the B cell compartment of the immune system. Additional linked T cell help does not overcome the poor 2F5 epitope immunogenicity in the homologous prime∶boost regimen involving either ES1 or ES2. These results indicate that the poor immunogenicity of the 2F5 epitope in context of these immunogens is not due to poor elicitation of T cell helper responses by these proteins. In contrast, the linked T cell help does increase the efficiency of heterologous prime∶boosting of the 2F5 epitope by ES1 and ES2, as long as ES5 first primes the 2F5 epitope-specific antibody/B cell responses. These data demonstrate that the non-immunogenic, ES2 (in terms of eliciting 2F5 epitope-specific responses), is capable of effectively driving 2F5 epitope-specific memory B cell responses if effectively primed. However, the magnitude of the 2F5 epitope-specific responses are not higher when elicited by the heterologous regimen compared to levels elicited by three homologous ES5+TH inoculations at either the serum antibody level or the B cell level, and were in fact lower than binding elicited by homologous ES5 prime∶boost lacking TH. Taken together, the data indicate that to achieve efficient heterologous prime∶boosting, it appears important, if not critical, to prime the response with an ES that efficiently presents the 2F5 epitope target to the immune system and to include linked T cell help in each immunogen.

There are some hints that the quality of the response subtly changes with heterologous prime∶boosting compared to the homologous ES5 immunization, suggesting that the heterologous scaffolding approach, when optimized, might be capable of influencing the specificities of a given B cell response. That there were slight improvements observed in the binding properties of the Mabs elicited by heterologous ES5 to ES1 prime∶boosting compared to ES5 homologous prime∶boosting, suggests that prime∶boosting may have some advantages if improvements in the immunogen design can be implemented. However, neutralization of HIV-1 was not elicited by any of the regimens tested in the study nor by any of the ES-elicited Mabs. Perhaps this is an issue of elicited antibody affinities for the bona fide, but as yet structurally undefined, 2F5 epitope in the native Env context. Or perhaps this is due to the lack of a lipid bilayer context in the immunogen. Eliciting antibodies similar to the parental 2F5 might require more hydrophobic surfaces to be present in the ES to drive the elicitation of antibodies capable of cross-reacting with the functional HIV-1 spike.

Why the ES5 protein presents the 2F5 epitope graft in a much more immunogenic manner in contrast to the other ES proteins tested here is unclear. In our previous study we showed a correlation between epitope flexibility and the ES capacity to generate epitope specific antibodies. Likely there are multiple factors involved that contribute to a better presentation of the epitope graft to the immune system. Is it that ES5 is a small scaffold with less (other) competing surface B cell epitopes or, similarly, that ES5 lacks competing immunodominant flexible loop epitopes? Or is it, perhaps, the oligomerization and occlusion of irrelevant scaffold epitopes? Studies to answer some of these questions are ongoing, such as “loosening” the graft in the ES2 context to determine the resulting biophysics and immunogenicity. This well-defined system highlights the different challenges between rendering a linear determinant (here) immunogenic, compared to applying this approach to a more complex conformational determinant such as the CD4 binding site of gp120 (recognized by the broadly neutralizing antibodies b12 and VRC01).

It seems unlikely, as has been suggested [54], that potential B cells recognizing the 2F5 epitope are deleted due to mimicry of some self epitope since, in the ES5 homologous immunization regimen and in the heterologous ES5-ES1-ES2 regimen there are substantial responses to the epitope. However, these ES were designed without regard to the undefined hydrophobic epitope contacts that presumably are made by the third complementarity-determining region of the heavy chain (CDRH3) of the antibody 2F5, which might be responsible for the self-specificity attributed to the antibody. Definition of these contacts of the 2F5 antibody might be necessary to further improve on the design of ES combinations capable of eliciting 2F5-like neutralizing antibodies.

In a recently published study, a similar scaffolding approach was utilized to present the poorly immunogenic gp41 4E10 epitope, which is located adjacent to the 2F5 epitope in the viral Env [42]. While the 4E10 ES elicited antibodies with an antigenic profile similar to the parental 4E10 monoclonal antibody, these 4E10 epitope-specific titers were quite low. The data presented here suggests that one way to potentially overcome the weak responses to the 4E10 epitope would be to adopt a heterologous prime∶boost strategy, perhaps by selecting the most immunogenic 4E10 ES as a prime and then boost with other 4E10 ES proteins that best mimic the epitope-bound conformation. In this scenario, as shown here, it would likely be important to include linked T cell help in the ES immunogens as was demonstrated in the current study for heterolgous ES prime∶boosting.

In a separate recent study, we demonstrated that the on-rate of ligands to the conformational gp120 co-receptor binding site is increased by conformational stabilization, resulting in an increase of antibodies targeted to the stabilized site [55]. These data suggested that perhaps conformational stabilization of a specific determinant might consistently enhance immunogenicity. In contrast, here, conformational fixation of the linear 2F5 epitope (by scaffolding) appears to decrease immunogenicity, at least in the ES2 context. Why this is so is not entirely clear. In part, there may be distinct B cell repertoire differences for the HIV-1 gp120 co-receptor binding site compared to the MPER. For the two sites, the virus may evade neutralization by one means completely different from the other, in part due to virus/Env fitness constraints. For the co-receptor site, HIV-1 evades the neutralization capacity of this antibody response by employing two receptors so that the CCR5 co-receptor site it is not exposed until after engagement of CD4 on the target cell. Therefore, it can tolerate avid responses to the occluded CCR5 binding site without a large cost to viral viability. However, to dampen responses to the presumably functionally conserved MPER, perhaps other means of immune evasion have been selected for in the host. The need by the virus to limit neutralizing antibodies to hydrophobic MPER was suggested previously to occur by some form of virus mimicry to ‘self’ human antigens [54].

In conclusion, effective responses to the engrafted 2F5 epitope were generated by means of a heterologous ES prime∶boosting immunization regimen. This effective prime∶boost response required priming with an immunogenic (to the epitope target) ES as well as linked T cell help via addition of the T cell helper epitope adduct in all ES immunogens. Non-immunogenic ES, but perhaps more loyal mimics of the targeted epitope can serve as boost proteins as long as priming is achieved with another ES. There is a need to make this process more efficient and capable of generating an HIV-1 neutralizing antibody response. In part, this might require the optimization of conformational fixation, while at the same time maintaining immunogenicity of the linear 2F5 epitope determinant.

## Supporting Information

Figure S1 **Epitope graft-specific titers.** Anti-ES4 titers (ES4 not utilized as immunogen) elicited with ES homologous immunization regimens. Panels on the top depict antibody responses of sera pooled from 5 mice prior to the first inoculation and after 1, 2 and 3 inoculations of ES protein immunogens lacking the heterologous T cell helper epitope (TH), and the bottom panels show responses elicited with TH-containing immunogens.
(TIF)
