# Supplementary figures and images for: Heterologous Epitope-Scaffold Prime∶Boosting Immuno-Focuses B Cell Responses to the HIV-1 gp41 2F5 Neutralization Determinant
Source: PLoS One. 2011 Jan 26;6(1):e16074. doi: 10.1371/journal.pone.0016074 (PMC3027617; doi:10.1371/journal.pone.0016074)

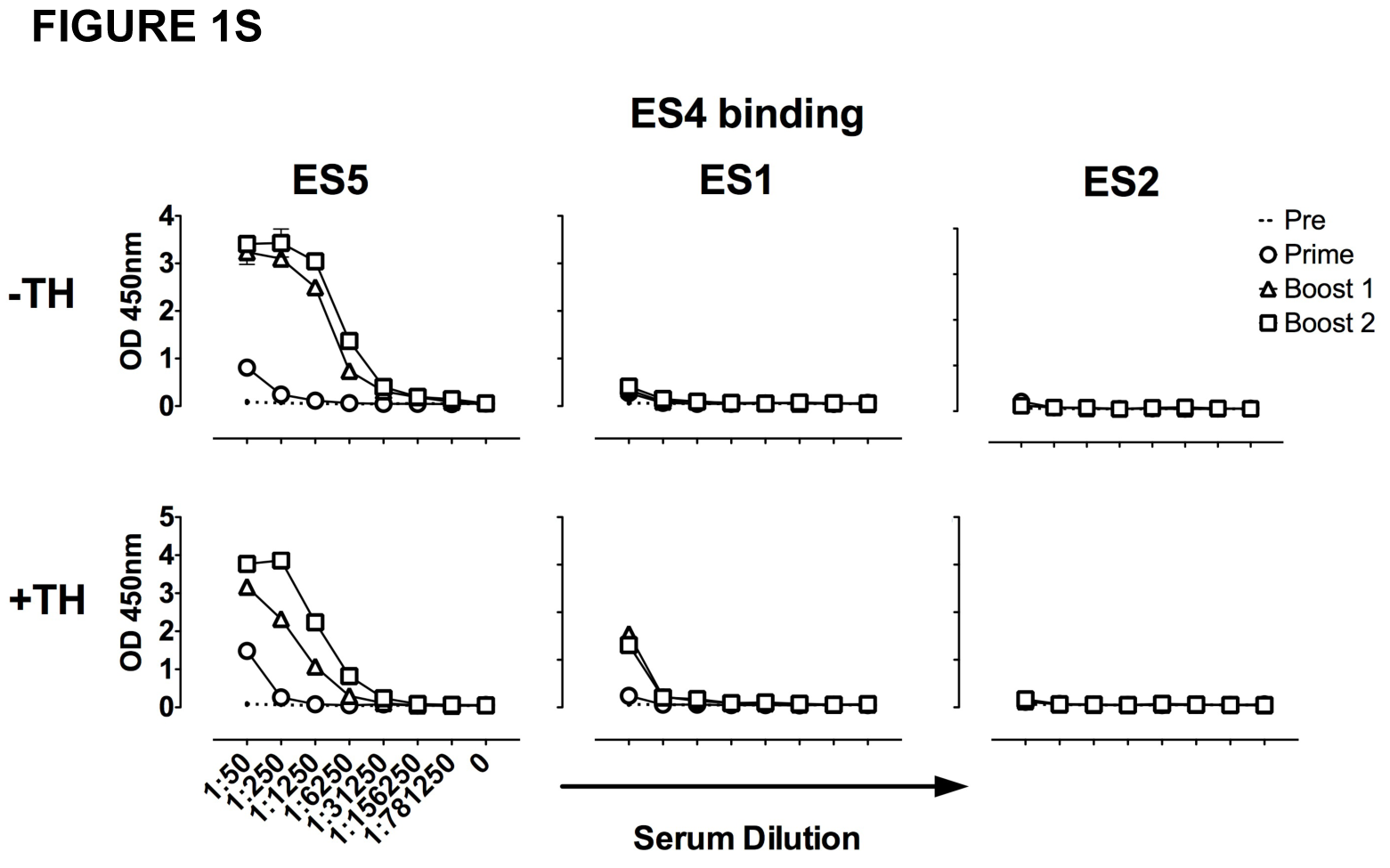

Supplement: Figure S1 — Epitope graft-specific titers. Anti-ES4 titers (ES4 not utilized as immunogen) elicited with ES homologous immunization regimens. Panels on the top depict antibody responses of sera pooled from 5 mice prior to the first inoculation and after 1, 2 and 3 inoculations of ES protein immunogens lacking the heterologous T cell helper epitope (TH), and the bottom panels show responses elicited with TH-containing immunogens. (TIF) [file pone.0016074.s001.tif]
